# Partial cor triatriatum sinistrum case series: is percutaneous balloon dilatation a promising alternative to surgery?

**DOI:** 10.1093/ehjcr/ytag215

**Published:** 2026-03-28

**Authors:** Lars S Witte, Abdelhak el Bouziani, Berto J Bouma, Frank van der Kley, David R Koolbergen, Danielle Robbers-Visser, Marcel A M Beijk, Robbert J de Winter

**Affiliations:** CAHAL, Center for Congenital Heart Disease Amsterdam-Leiden, Albinusdreef 2, Leiden 2333 ZA, The Netherlands; Department of Cardiology, Amsterdam University Medical Centers, Meibergdreef 9, Amsterdam 1105 AZ, The Netherlands; CAHAL, Center for Congenital Heart Disease Amsterdam-Leiden,Albinusdreef 2, Leiden 2333 ZA, The Netherlands; Department of Cardiology, Amsterdam University Medical Centers, Meibergdreef 9, Amsterdam 1105 AZ, The Netherlands; CAHAL, Center for Congenital Heart Disease Amsterdam-Leiden,Albinusdreef 2, Leiden 2333 ZA, The Netherlands; Department of Cardiology, Amsterdam University Medical Centers, Meibergdreef 9, Amsterdam 1105 AZ, The Netherlands; CAHAL, Center for Congenital Heart Disease Amsterdam-Leiden,Albinusdreef 2, Leiden 2333 ZA, The Netherlands; Department of Cardiology, Leiden University Medical Center, Albinusdreef 2, Leiden 2333 ZA, The Netherlands; CAHAL, Center for Congenital Heart Disease Amsterdam-Leiden,Albinusdreef 2, Leiden 2333 ZA, The Netherlands; Department of Congenital Cardiothoracic Surgery, Leiden University Medical Center, Albinusdreef 2, Leiden 2333 ZA, The Netherlands; CAHAL, Center for Congenital Heart Disease Amsterdam-Leiden,Albinusdreef 2, Leiden 2333 ZA, The Netherlands; Department of Cardiology, Amsterdam University Medical Centers, Meibergdreef 9, Amsterdam 1105 AZ, The Netherlands; CAHAL, Center for Congenital Heart Disease Amsterdam-Leiden,Albinusdreef 2, Leiden 2333 ZA, The Netherlands; Department of Cardiology, Amsterdam University Medical Centers, Meibergdreef 9, Amsterdam 1105 AZ, The Netherlands; CAHAL, Center for Congenital Heart Disease Amsterdam-Leiden,Albinusdreef 2, Leiden 2333 ZA, The Netherlands; Department of Cardiology, Amsterdam University Medical Centers, Meibergdreef 9, Amsterdam 1105 AZ, The Netherlands

**Keywords:** Cor triatriatum, Percutaneous, Congenital heart disease, Case series

## Abstract

**Background:**

Partial cor triatriatum sinistrum is a rare congenital heart disease and is usually considered for surgery in symptomatic patients. We describe three cases of partial cor triatriatum sinistrum, two cases of successful percutaneous balloon dilatation, and one case of conservative treatment in a patient without symptoms.

**Case summary:**

The first case describes a female patient with progressive dyspnoea on exertion in which the membrane was dilated with a balloon to relieve symptoms. The second case is about a young patient with chronic congestion of the right lung because of a partial cor triatriatum sinistrum which was also treated with dilatation. The third case describes a male patient with an incidental finding of a cor triatriatum sinistrum who did not experience any symptoms and was treated conservatively.

**Discussion:**

Percutaneous balloon dilatation is a safe and effective alternative to surgery in selected cases of (partial) cor triatriatum.

Learning points

- Partial cor triatriatum can be diagnosed at advanced age, and clinical manifestations can develop slowly.
- Percutaneous balloon dilatation for partial cor triatriatum is a promising alternative when surgical resection is deemed too invasive in patients with favourable anatomy and without other cardiac abnormalities.

## Introduction

Cor triatriatum sinistrum is a rare congenital heart disease with multiple anatomical variations, and it is present in <0.1%–0.4% of all congenital heart disease.^1,2^ The classic variant of cor triatriatum sinistrum is characterized by an accessory chamber receiving all pulmonary venous return and is separated from the left atrium by a fenestrated membrane or tubular connection.^3^ In the partial variant of cor triatriatum, the accessory chamber only receives part of the pulmonary venous return. If the communication via the fenestrated membrane is small, it results in obstruction of pulmonary venous drainage. In partial cor triatriatum, this can lead to unilateral pulmonary venous congestion and pulmonary hypertension.

To date, (partial) cor triatriatum with an obstructive membrane leading to symptoms is usually considered for surgery.^4^ We have previously shown in a small surgical series that balloon dilatation of the membrane seems feasible and could be considered as an alternative to surgery.^5^ In a few selected cases, some recently published percutaneous treatment options were shown to be effective and safe, although long-term outcomes remain unclear.^6–9^ Whether partial cor triatriatum sinistra are accessible for percutaneous balloon dilatation is unknown. We describe two cases of successful percutaneous balloon dilatation of partial cor triatriatum sinistra, suggesting the safety and effectiveness of this percutaneous approach as an alternative to surgery. Additionally, we present a case of an incidental finding of cor triatriatum sinistrum which was treated conservatively.

## Summary figure

**Figure:**
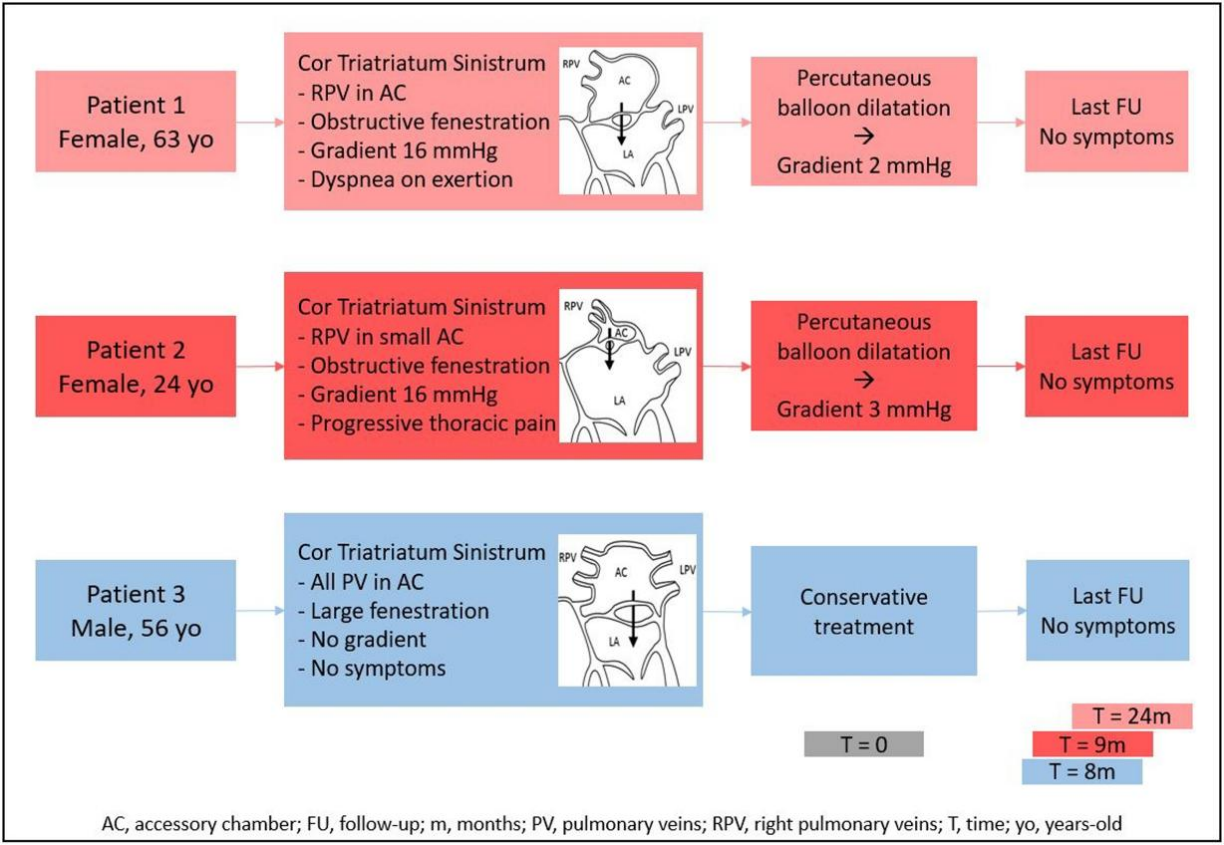


## Procedure

We performed two cases of percutaneous balloon dilatation of partial cor triatriatum sinistra, modified classification according to Lucas Type IIIA1 (subtotal cor triatriatum in which the accessory atrial chamber receives part of the pulmonary veins and connects to the left atrium, the remaining pulmonary veins connect normally). Procedures were performed under general anaesthesia with transoesophageal echocardiography (TEE) guidance. Access was achieved via the right femoral vein and artery with an 11 and 6 Fr guide, respectively. Transseptal puncture was performed using standard techniques (BKR needle) with TEE guidance.

## Patient 1

The first case is a 63-year-old female with a history of a cerebrovascular accident without sequelae, hypothyroidism, and migraine. She suffered from COVID-19 pneumonia for which she was not hospitalized, but recuperated at home. After the pneumonia, she experienced prolonged symptoms which were initially assumed to be long COVID. Furthermore, she experienced progressive dyspnoea on exertion, which in hindsight started several years before the pneumonia. Physical examination at presentation was unremarkable.

A computed tomography scan was made by the pulmonologist and revealed a contrast-negative structure in the left atrium, suspect for myxoma, lymphoma, or cor triatriatum.^10^ Additional magnetic resonance imaging confirmed a cor triatriatum sinistrum with restricted drainage of solely both right pulmonary veins into the accessory (proximal) atrial chamber and an obstructive fenestrated membrane to the distal left atrium. Transthoracic and transoesophageal echocardiography revealed a gradient of 16 mmHg over the membrane and no pulmonary hypertension.

The patient had no pulmonary hypertension or left-sided heart failure but prolonged complaints of dyspnoea on exertion and restricted drainage of the right pulmonary veins with perfusion mismatch. Surgical resection was considered to be unattractive since the expected benefit in the reduction of symptoms was uncertain. Therefore, less invasive percutaneous treatment was decided.

**Figure 1:**
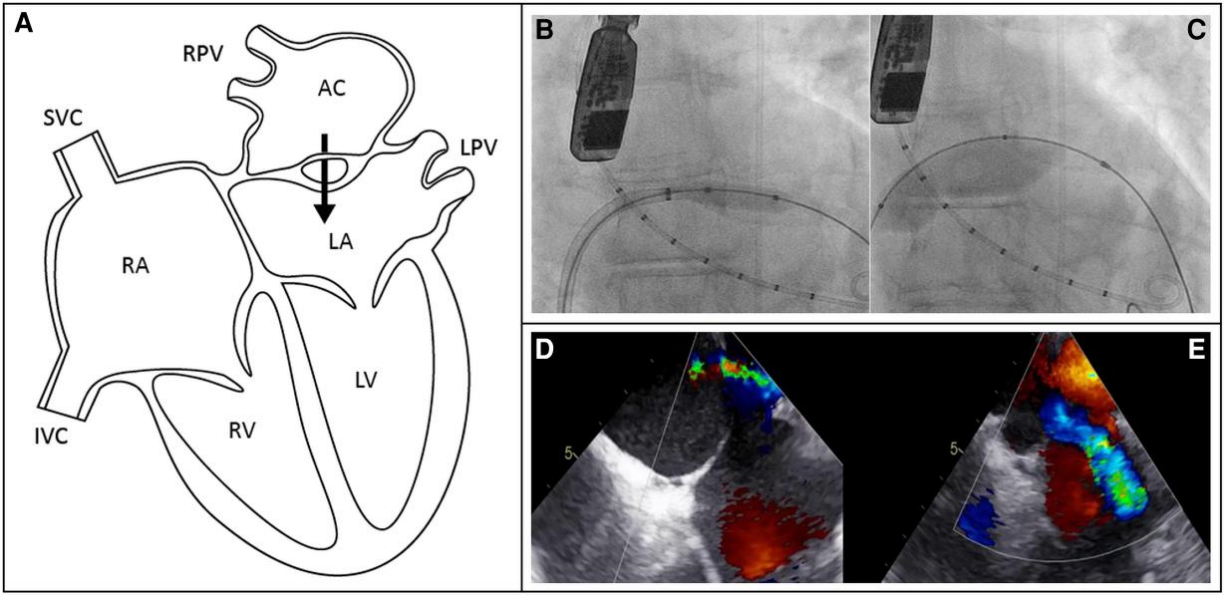
Anatomy, angiography, and echocardiography of patient 1. *(A)* Illustration of the anatomy of the partial cor triatriatum sinistrum with a fenestrated accessory chamber. *(B, C)* Procedural angiography of the balloon dilatation of the membrane. *(D)* The TEE pre-procedural flow through the fenestrated membrane. *(E)* The TEE post-procedural flow over the membrane after balloon dilatation. AC, accessory chamber; IVC, inferior vena cava; LA, left atrium; LPV, left pulmonary veins; LV, left ventricle; RA, right atrium; RPV, right pulmonary veins; RV, right ventricle; SVC, superior vena cava.

**Figure 2:**
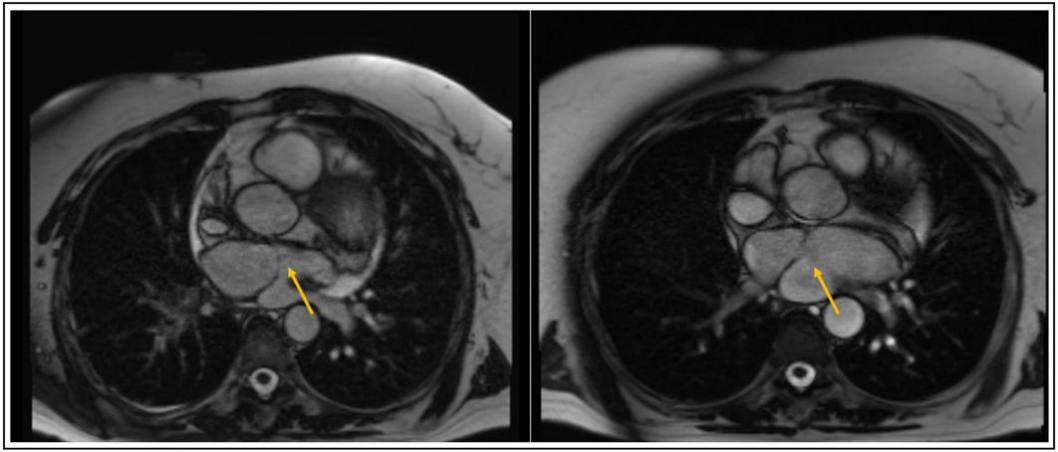
Magnetic resonance imaging of Patient 1 before and after percutaneous balloon dilatation. On the left, a large accessory chamber is seen with a small, restricted opening across the membrane to the left atrium. On the right, 18 months after percutaneous balloon dilatation, a decrease in the dimension of the accessory chamber is seen with an increased opening across the membrane to the left atrium.

Under general anaesthesia and TEE guidance, access was obtained across the atrial septum to the posterior chamber using a Baylis wire, and an Agilis steerable guiding was positioned. The gradient over the cor triatriatum membrane was measured as 16 mmHg (under anaesthesia), pulmonary pressures were borderline [mean pressure (mmHg): pulmonary artery 25; right ventricle 18; right atrium 11; right upper pulmonary vein 24; left atrium 10]. With a 6 Fr guiding catheter, a microcatheter, and the Baylis RF wire, under TEE guidance the membrane was crossed at a central location, after which a stiff Amplatz wire was passed across the membrane. Subsequently, the membrane was stepwise dilated up to a 25 mm balloon (at 4 atm). A central defect in the membrane was created, measuring approximately 12 mm in diameter on TEE (*Figure 1*). After the procedure, the pressure gradient over the membrane decreased from 16 to 2 mmHg. Twenty-four months later, transthoracic echocardiography showed a pressure gradient of 2 mmHg, and the patient’s symptoms were completely resolved. Additional magnetic resonance imaging showed decreased dimensions of the accessory chamber which filled simultaneously with the left atrium, and the right lung perfusion was improved (*Figure 2*).

## Patient 2

The second case is a 24-year-old female with a history of asthma. She suffered from episodes of stabbing right thoracic pain, partially evoked by exertion. Recently, the episodes of pain became more frequent, and compared to her peers, she experienced early shortness of breath during exercise for which the treating pulmonologist eventually performed a computed tomography scan. Physical examination at presentation was unremarkable.

The computed tomography scan revealed decreased perfusion of the right lung, a small right pulmonary artery, unilateral atresia of the right pulmonary vein, and congestion of the right lung. The cardiology department was consulted, and a cardiac magnetic resonance imaging was made which revealed a partial cor triatriatum sinistrum with restricted drainage of both right pulmonary veins with a stenotic common ostium into the accessory atrial chamber. Close by the common ostium was a fenestrated membrane to the distal left atrium. Additional transthoracic and transoesophageal echocardiography showed a gradient of 16 mmHg over the membrane.

Since this young patient had chronic congestion of the right lung, interventional treatment was advised. After shared decision-making with the patient, percutaneous treatment was performed.

**Figure 3:**
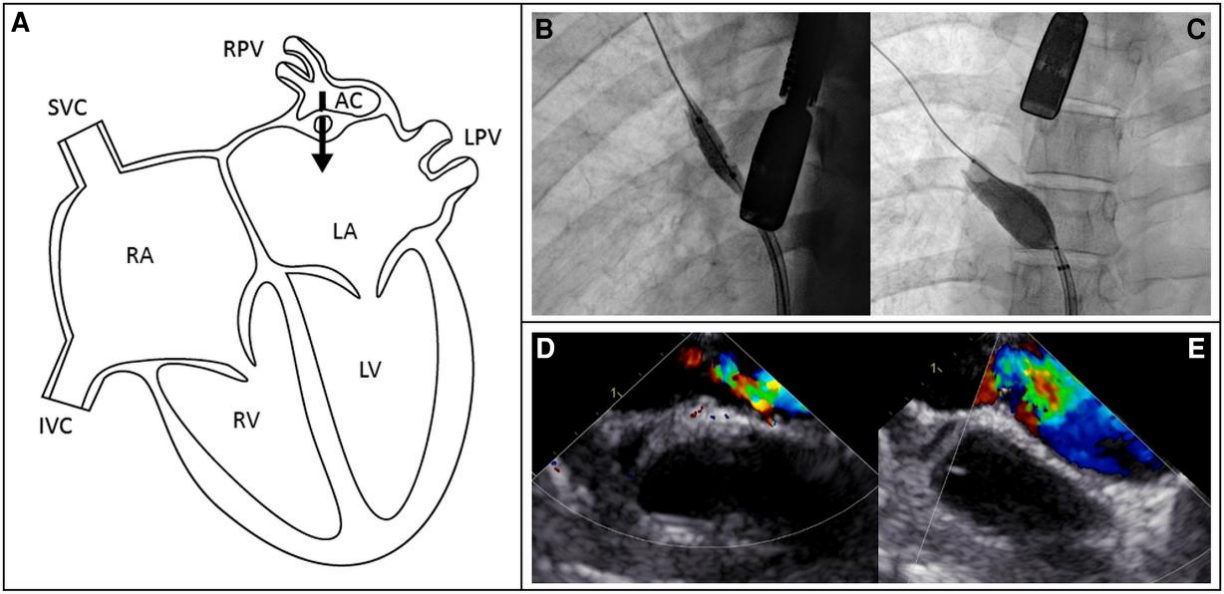
Anatomy, angiography, and echocardiography of Patient 2. *(A)* Illustration of the anatomy of the partial cor triatriatum sinistrum with a small fenestrated accessory chamber close to the hypoplastic right pulmonary veins. *(B, C)* Procedural angiography of the balloon dilatation of the membrane. *(D)* The TEE pre-procedural flow through the fenestrated membrane. *(E)* TEE post-procedural flow over membrane after balloon dilatation. AC, accessory chamber; IVC, inferior vena cava; LA, left atrium; LPV, left pulmonary veins; LV, left ventricle; RA, right atrium; RPV, right pulmonary veins; RV, right ventricle; SVC, superior vena cava.

The patient underwent TEE-guided percutaneous balloon dilatation of the fenestrated membrane of the cor triatriatum. A standard transseptal puncture was performed, and an Agilis steerable sheath was advanced into the left atrium. With a second venous catheter, contrast was injected into the right pulmonary artery, which showed an upper and middle pulmonary vein joining and showing a contrast jet into the left atrium. In addition, transthoracic echocardiography showed the connection across the membrane to be centrally located. Pressures were measured that showed a pressure gradient of 15 mmHg over the cor triatriatum membrane without the presence of pulmonary hypertension [mean pressure (mmHg): pulmonary artery 20; right ventricle 8; right atrium 5; right upper pulmonary vein 21; left atrium 6]. The membrane was crossed retrogradely with a wire, and the membrane was gradually dilated up to a 16 mm balloon (at 4 atm) (*Figure 3*). At the end of the procedure, the pressure gradient over the membrane had decreased from 15 to 3 mmHg. The patient is now 9 months after the procedure and has improved.

## Patient 3

**Figure 4:**
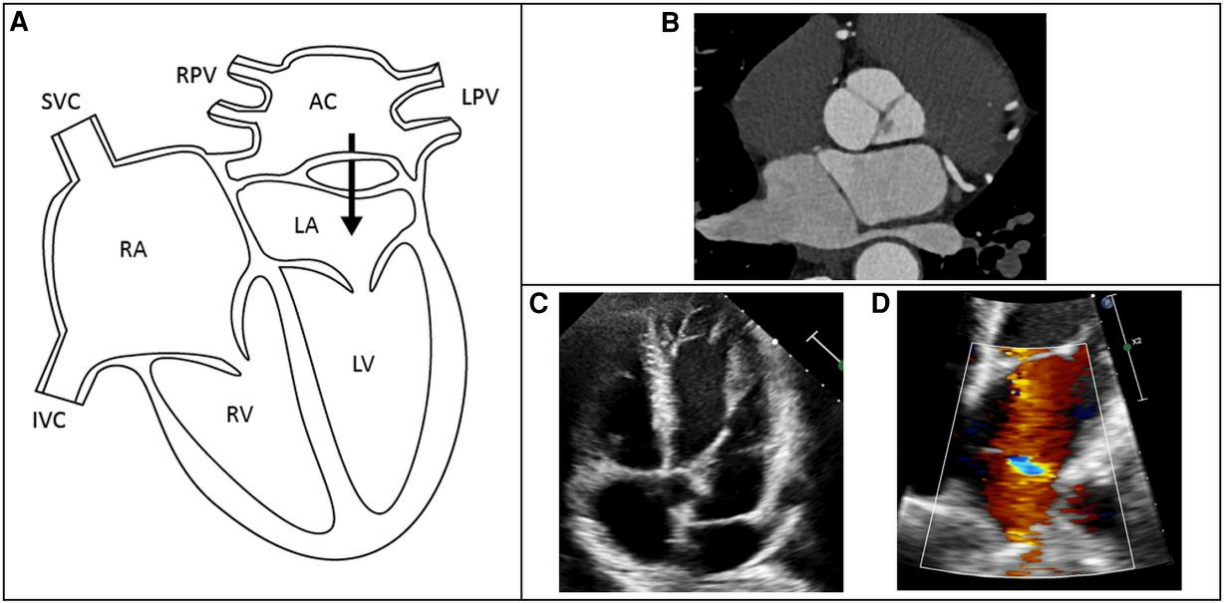
Anatomy, computed tomography, and echocardiography of Patient 3. *(A)* Illustration of the anatomy of the cor triatriatum sinistrum with a large fenestrated accessory chamber. *(B)* Computed tomography with all pulmonary veins draining into the accessory chamber. *(C, D)* Transthoracic echocardiography of the cor triatriatum with flow through the fenestration. AC, accessory chamber; IVC, inferior vena cava; LA, left atrium; LPV, left pulmonary veins; LV, left ventricle; RA, right atrium; RPV, right pulmonary veins; RV, right ventricle; SVC, superior vena cava.

The third case is a 56-year-old male with no remarkable history. He experienced no symptoms, and physical examination was unremarkable, but he had an increased cardiac risk profile because of family history. Therefore, he underwent cardiac screening including a computed tomography scan, which revealed an incidental finding of a cor triatriatum sinistrum. In this case, all pulmonary veins drained unrestrictedly into the accessory chamber (modified classification according to Lucas Type 1A) (*Figure 4*). The membrane had a large fenestration of 36 × 29 mm without a gradient. Given the absence of symptoms and the notably large fenestration without any signs of lung congestion, the decision was made to manage the cor triatriatum conservatively. Eight months later, the patient was still without any symptoms.

## Discussion

To our knowledge, this is the first report of two cases of successful percutaneous balloon dilatation in isolated partial cor triatriatum sinistrum resulting in relief of restrictive right pulmonary venous drainage from the accessory to left atrial chamber. The current evidence on percutaneous treatment for partial cor triatriatum sinistrum is lacking, and there is only a handful of case reports on classic cor triatriatum sinistrum.^6–9^ The mechanism of persistent opening of the membrane after balloon dilatation has not been studied yet. However, this might be because of small ruptures of the membrane by dilating the balloon, which increases free flow over the membrane and prevents recoil over time. According to the American College of Cardiology and American Heart Association guidelines, surgery is the preferred treatment option for cor triatriatum based on three small observational studies that included 15, 25, and 65 patients.^4,11–13^ To date, there are no European guidelines on the management of cor triatriatum.

Balloon dilatation has been used as a bridge to definitive surgical treatment in children and adults with cor triatriatum and as definitive treatment in cor triatriatum dexter, after assessment of anatomical characteristics and in the absence of other cardiac abnormalities.^14^ When surgical treatment is deemed to be high-risk, percutaneous treatment with balloon dilatation has proven to be a successful alternative.^15^ Furthermore, in cor triatriatum dexter, percutaneous treatment has been demonstrated to be effective in multiple cases, indicating that balloon dilatation could be an alternative to surgery in selected cases of (partial) cor triatriatum sinistrum as well.^16–18^

A previous report on hybrid treatment of balloon dilatation combined with surgery to treat cor triatriatum showed the feasibility of percutaneous treatment options, with the remarks of suited anatomy (all pulmonary veins must ultimately drain into the left atrium, Types IA and IIIA1) and the absence of other cardiac abnormalities requiring surgery.^5^ This emphasizes the importance of detailed imaging and evaluation to help understand the anatomy of cor triatriata for optimal exploration of possible treatment options, including conservative treatment.^19^

Nowadays, new techniques, equipment, and imaging modalities to select individual cases suited for percutaneous treatment are available. As shown in previous reports, percutaneous treatment is safe and effective in cor triatriatum sinistrum and dexter. With this report of successful treatment of partial cor triatriatum with percutaneous balloon dilatation, the potential of percutaneous treatment options in the broad anatomical variance of cor triatriatum is further expanded.

In conclusion, cor triatriatum is a very rare condition, and there is limited evidence concerning treatment consisting of case reports or small series. Current guidelines are based on small observational studies of surgical treatment, wherein most patients had additional cardiac abnormalities requiring concomitant surgical treatment. As demonstrated by our cases and prior reports, in selected cases of (partial) cor triatriatum, percutaneous balloon dilatation is a safe and effective alternative to surgery, especially in cases of diagnosis at advanced age and in the absence of other cardiac abnormalities. Our first case is now 24 months after the procedure and shows no sign of recurrence of the gradient over the membrane. Long-term follow-up is needed to ensure that percutaneous treatment is a lasting alternative to surgery.

## Data Availability

The data underlying this article will be shared on reasonable request to the corresponding author.

## Lead author biography

Lars S. Witte completed his study in medicine at the Vrije Universiteit van Amsterdam, The Netherlands, in 2019 and is currently a medical doctor and PhD candidate at the University of Amsterdam Department of Cardiology.
